# Assessing climate change effects on Turkish tea farming through a dual approach using MMQR and machine learning

**DOI:** 10.1038/s41598-025-29358-8

**Published:** 2025-12-16

**Authors:** Tunahan Haciimamoglu, Gokan Bulbul, Korkmaz Yildirim, Burcu Kartal

**Affiliations:** 1 Department of Economics, Faculty of Economics and Administrative Sciences, Recep Tayyip Erdoğan University, Fener Street, Zihni Derin Campus, Rize, 53100 Türkiye; 2 Master’s Student, Institute of Graduate Studies, Recep Tayyip Erdoğan University, Rize, 53100 Türkiye; 3 Department of Political Science and Public Administration, Faculty of Economics and Administrative Sciences, Recep Tayyip Erdoğan University, Rize, 53100 Türkiye; 4 Department of Business Administration, Faculty of Economics and Administrative Sciences, Recep Tayyip Erdoğan University, Rize, 53100 Türkiye

**Keywords:** Sustainable farming, Tea agronomy, Climate adaptation, Ensemble learning, Method of moments quantile regression, Climate-change impacts, Agroecology, Climate-change ecology

## Abstract

Climate change increasingly threatens the productivity of region-specific strategic agricultural products such as tea cultivation in Türkiye, posing a serious risk to both food security and rural economies. However, existing literature is notably limited in terms of studies that draw attention to this risk and examine the effects of climate change on tea productivity at a regional scale through rigorous quantitative methods. To this end, this study investigates the influence of climate change on tea productivity in Türkiye’s tea–growing provinces (Artvin, Giresun, Ordu, Rize, and Trabzon) between 2004 and 2022. Distinct from previous studies, we integrate advanced machine learning techniques with the method of moments quantile regression (MMQR) approach to provide comprehensive, reliable, and methodologically robust results for the first time in this context. The results of the MMQR demonstrate that although humidity reduces tea productivity, temperature and precipitation significantly increase it. Furthermore, the results of machine learning research indicate that the tea farming area is the variable with the highest importance, whereas humidity emerges as the least influential factor. These findings indicate that policymakers need to implement integrated agricultural policies in the five tea–growing provinces of the Eastern Black Sea region, including effective moisture management, soil fertility, erosion control, and irrigation infrastructure tailored to the climate and land conditions.

**Supplementary Information:**

The online version contains supplementary material available at 10.1038/s41598-025-29358-8.

## Introduction

Since the early 20th century, drastic and accelerating changes in the global climate system have caused substantial losses in agricultural production and serious disruptions in the food supply chain, affecting most regions of the world^1,2^. For example, food security^3^, groundwater resources^4^, renewable energy sources^5^, agriculture and livestock^6–9^, nature tourism^10^, cultural heritage^11^, forestry^12^, and vector–borne diseases^13^ are just a few of the many ecosystems threatened by the adverse effects of global climate change. At the national level, declining agricultural yields and productivity loss brought on by the adverse effects of climate change exacerbate economic and social inequality^14^. Yet, despite the growing global evidence, the localized consequences of climate change on regional economies and ecosystems remain underexplored, particularly in vulnerable sectors such as agriculture.

This gap underscores the need for a more comprehensive examination of human activities that shape the climate system. Specifically, because of intensive human production and consumption activities such as burning fossil fuels, rapid urbanization, and industrialization, the global rate of greenhouse gas emissions has been continuously rising. Such changes have led to more extreme weather, higher temperatures, irregular rainfall, and increased erosion across the globe. Over time, these factors progressively lower agricultural yields, which in turn has a detrimental impact on rural development and economic sustainability^15^. It is not known exactly whether this is the case for tea production, but climate change has nonetheless become a critical global issue^16^. Because of climate change, extreme weather events such as storms, floods, inconsistent precipitation, and rising average temperatures have a major detrimental impact on agricultural activities, which are crucial to the economies of developing nations^17^. One of the main factors slowing economic growth and endangering sustainable rural development is the mounting strain that climate change places on agricultural activities^18^, so researchers should also examine climate change impacts at the regional and local levels for strategically important agricultural crops such as tea. Tea is one such crop, particularly in the Eastern Black Sea Region of Türkiye, where the majority of national tea production occurs. Additionally, tea productivity is highly sensitive to changes in temperature, rainfall, and humidity, which are climatic factors that are being increasingly destabilized.

However, this sensitivity has not been adequately addressed in the literature. Indeed, the majority of studies have focused on the global or national-level impact of climate change and have paid relatively little attention to its effects on tea farming, particularly at the regional level^19^. However, because of climatic and geographic limitations, changes are nonetheless noticeable in tea-producing countries such as China, India, Kenya, and Türkiye^20,21^. Tea is a strategically vital source of income for these countries and is one of the most consumed national and global agricultural products. For example, global tea production reaches approximately 30 million tons annually. Türkiye, where production capacity is concentrated in the Black Sea region, is among the top five tea-producing countries worldwide^22^. The 207 tea factories located solely in the Black Sea region produce approximately 19,000 tons of tea daily^23^. The tea sector plays a critical role in economic and rural development, poverty reduction, and food security, providing income and employment for millions of families, especially in low-income countries^24^. Given this high socio-economic and agricultural importance, the vulnerability of tea farming to climate variability poses a direct threat to livelihoods, regional trade, and food security. Thus, there remains a significant research gap in understanding these dynamics within the regional context in Türkiye.

On the other hand, tea production worldwide has recorded a remarkable growth in recent years. Global tea production has exhibited steady growth over the past decades. According to FAO data, global tea production reached approximately 6.7 million metric tons in 2023, representing an average annual growth rate of about 3.2% over the past decade. The largest producers are China (≈ 48% of world output), India (≈ 22%), Kenya (≈ 8%), Sri Lanka (≈ 5%), and Türkiye (≈ 4%). Türkiye is both a significant producer and the world leader in per capita tea consumption. In 2019, the country produced approximately 1.45 million tons of fresh tea leaves, corresponding to about 260–270 thousand tons of processed tea. In 2023, the country produced 343,500 tons of processed tea, primarily for domestic consumption. Per capita consumption in Türkiye is the highest in the world, estimated at 3.16–4.6 kg annually, depending on the source and year. The majority of production occurs in the Eastern Black Sea region, particularly in Rize, Trabzon, Artvin, and Giresun provinces, with Rize alone accounting for more than 60% of the cultivation area. Although domestic consumption is dominant, Türkiye also exports to over 100 countries, with significant markets in the European Union, Russia, and the Middle East^22,25,26^. In terms of consumption, tea remains the most consumed beverage worldwide after water. The global tea market was valued at USD 25.6 billion in 2024 and is projected to reach USD 38.1 billion by 2033 (CAGR ≈ 4.5%). The data from recent years show that global tea consumption expanded by 2% in 2022 compared to 2021, driven by high growth in producing countries^27^. Per capita consumption varies significantly: Türkiye, Ireland, and the United Kingdom lead globally with annual consumption often exceeding 3 kg per person, while large consuming markets such as China and India have lower per capita figures but very high aggregate demand^22,28,29^.

In light of these global market dynamics, the sensitivity of tea production in Türkiye’s Eastern Black Sea Region to climate change merits examination. To this end, this study investigates the effects of climate change on regional and local tea productivity in the provinces of Artvin, Giresun, Ordu, Rize, and Trabzon. The strategic and economic significance of the Eastern Black Sea Region in tea production and its dominant position in worldwide tea output serve as our primary motivation. Tea production in Türkiye has increased from 1,442 kg/day in 2004 to 1,563 kg/day in 2022, positioning it as one of the world’s top tea-producing countries. However, a deeper understanding of the detrimental effects of climate change on local and regional agriculture is necessary to ensure agricultural sustainability. More research is needed on the effects of climate change on regional and local tea farming, given its strategic social and economic importance to the economy and society of the Eastern Black Sea Region. In addition to showcasing the development of sustainable agricultural practices, the tea sector is also a critical source of revenue for farmers and the local population. Addressing this gap, this study aims to answer key questions such as: How sensitive is tea production in Türkiye to climate variability? Which climatic factors have the most influence on regional tea yields? How can we methodologically measure and analyze these impacts with precision?

This study is unique in several aspects. First, this study is, to our knowledge, the first empirical investigation to assess how climate change affects the tea productivity in Türkiye—a key agricultural product with strong socio–economic ramifications in the Black Sea Region. By focusing on the provinces of Artvin, Giresun, Ordu, Rize, and Trabzon in the region, this study uniquely captures the subnational and region-specific dynamics of climate change’s impact on agricultural productivity, offering insights that are currently lacking in the literature. Second, the study also contributes novel perspectives on climate adaptation strategies by focusing specifically on tea farming in the region. Unlike many other crops, there has been a body of evidence that tea is particularly sensitive to climate change. So, by examining how these factors influence tea productivity in the region, this study provides practical recommendations for local climate adaptation, which are crucial for stakeholders in the region, such as farmers and policymakers. Third, distinct from prior empirical research, this study employs a complementary dual methodological approach that uniquely combines the method of moments quantile regression (MMQR) with advanced machine learning techniques (Gradient Boosting (GB), Extreme Gradient Boosting (XGBoost), and Genetic Algorithm-Extreme Gradient Boosting (GA-XGBoost)). MMQR enables us to examine how climate variables affect tea productivity differently across the yield distribution (i.e., in low-yielding vs. high-yielding provinces), revealing heterogeneous effects that conventional OLS regression cannot capture. However, regression-based approaches, including MMQR, cannot determine the relative importance of explanatory variables. Machine learning models, in turn, identify which climatic and non-climatic variables are most important for overall predictive accuracy and uncover potential nonlinear relationships and interactions. Together, this dual approach provides a more complete analytical picture. This integrated framework offers novel, policy-relevant insights that neither method alone could deliver, making it particularly valuable for designing targeted adaptation strategies.

We have organized the sections of the study as follows. In the second section we explain the theoretical framework, and comprises literature reviews of the variables. In Sect. 3, we introduce the model and dataset, and in Sect. 4, we describe the estimating approaches. The findings and discussion appear in Sect. 5. In Sect. 6, we present the study’s findings and policy recommendations.

## Theoretical background and literature review

Climate change refers to long–term shifts in climate characteristics on a global or regional scale caused by both natural and anthropogenic forces. Based on comprehensive scientific reports, one of the primary causes of global climate change is the sharp rise in greenhouse gas emissions following the industrial revolution^30^. Understanding sectoral adaptation and the actions needed to counteract these changes is crucial for managing the potential influence of climate change^31^. To this end, sustainable agricultural practices are increasingly inevitable. Furthermore, incorporating cutting–edge and climate-resilient methods of agricultural production and management into conventional farming practices in light of climate projections is essential for sustainable agriculture^32^. In agricultural practices that are susceptible to the adverse consequences of climate change, policymakers should employ adaptive and mitigative strategies to boost resilience and productivity^33^. In fact, because the agricultural sector is more vulnerable to the consequences of climate drivers than other sectors, extreme weather events such as droughts and floods have especially severe and long-lasting detrimental impact on productivity and output^34^.

Because agricultural sustainability is a multidimensional and complicated topic, it merits assessment at several levels, including local, national, and global^35^. Indeed, the ambiguities and debates around sustainability are the foundation of this concept’s complexity^36^. Agricultural sustainability is also a holistic and integrative approach that emphasizes environmental and natural resource preservation while requiring the use of suitable agricultural practices to boost productivity to satisfy the world’s growing food demand^37^. This strategy not only has positive effects on food and nutritional security but also contributes to the adoption of innovative agricultural approaches on a global scale, helping agricultural sectors worldwide to achieve a more resilient and sustainable structure^38^.

### The nexus of climate change, agricultural activities, and economic growth

Scholarly debates have centered on the nexus among economic growth, agricultural sustainability, and extreme weather occurrences such as droughts and high temperatures brought on by global climate change, particularly in the post-COVID–19 period. For example, from an economic point of view, Bilal and Kanzig^39^ proposed that a 1 °C rise in temperature might result in a 12% decline in world GDP. This estimate shows the potential for larger losses and the significant correlation between extreme climate events and the global economy. In their analysis of 174 nations from 1960 to 2014, Kahn et al.^40^ also calculated that a 0.04 °C increase in temperature would have a detrimental impact on economic growth, with losses worldwide potentially reaching 13% by 2100. Similarly, according to Lemaire^41^, a consistent 1 °C rise in temperature in developing nations lowers the rate of economic growth by 1.25%. Finally, Byrne and Vitenu–Sackey^42^ also argued that both developed and developing nations’ economic and socioeconomic indicators suffer from rising temperatures brought on by climate change. Based on the findings of this study, rising temperatures have a more pronounced and disproportionate impact on economic growth, particularly in developing countries.

There is increasing evidence that climate change, through erosion, erratic precipitation, and rising average temperatures, reduces agricultural productivity, which in turn hampers economic growth^17,18^. Production declines as a result of productivity loss, and the cost of associated agricultural products rises accordingly. These consequences are becoming more noticeable and disruptive in countries where agriculture is the primary economic sector. Thus, both economic growth and agricultural practice in these countries more clearly show the direct and measurable impact of climate change. For example, Trinh^43^ found that climate change negatively affects farm net income, a crucial measure of agricultural production. Both developed and developing countries experience these effects. Adams et al.^44^ also assessed how climate change was affecting the U.S. agricultural sector and confirmed that temperature increases and fluctuating rainfall affect agricultural yields, with significant implications for global food security. Similarly, Lu et al.^45^ determined that climate change has a detrimental impact on agricultural yields, particularly because of temperature increases and water resource constraints, without looking at how it affects grain production. Burke et al.^31^ also demonstrated that excessive heat reduces agricultural yields, thereby endangering global production. Additionally, in their study spanning the years 2000–2019, Dubovitski et al.^46^ examined climate threats to Russia’s agricultural industry in the context of climate change. The results indicated that annual temperature fluctuations and increased precipitation lead to yield losses.

In addition to the agricultural sector, global climate change also directly affects economic growth. In this regard, Fankhauser and Tol^47^ highlighted how labour, savings, and capital accumulation are all negatively affected by climate change. In a similar vein, Dell et al.^48^ discovered that a 1 °C rise in temperature in developing countries between 1950 and 2003 slowed economic growth by 1.1%. Their report also noted that although the consequences of rising temperatures are more pronounced in low-income countries, they are less so in wealthy ones. Similarly, Raddatz^49^ claimed that low–income countries are particularly vulnerable to climate change and that it reduces economic development by about 1%. In addition, the economic effects of climate change in middle- and high-income countries are more limited and generally vary between 0.25% and 0.5%. On this issue, Tol^50^ also evaluated the political and economic effects of climate change and concluded that, despite its initial benefits over the past century, it has had a longer-term negative impact on the economy and well-being of countries with lower incomes and higher temperatures.

### The interlinkages of climate change and tea production

As climate change impacts vary significantly across regions and crop types, understanding how these global patterns translate into specific agricultural contexts is essential. Tea cultivation, being both strategically vital and highly sensitive to climatic variability, offers an ideal case for examining localized impacts and adaptation challenges.

Building on this understanding, researchers have recently focused more on the crop-specific consequences of climate change for tea farming, conducting empirical studies using samples from various countries. However, there is currently little consensus in the literature regarding how climate change affects tea productivity. Wijeratne^51^ provided empirical evidence of the beneficial impacts of increased warmth and rainfall on Sri Lankan tea agricultural yield. Biggs et al.^52^, however, stressed that this is not the case for other nations and claimed that India’s tea production was adversely affected by rising temperatures and fluctuating rainfall. Similarly, Mallik and Ghosh^53^ investigated how climate change affected tea production in India, finding that high temperatures and erratic rainfall had detrimental impacts. Furthermore, Lou et al.^54^ explored how climate change impacted tea output from 1985 to 2018 in China and found that drought and high temperatures had a detrimental impact on tea productivity. These findings were consistent with previous studies. For example, Cheserek et al.^55^ investigated the connection between Kenyan tea yield and temperature, showing that raising the average air temperature by up to 19.2 °C enhanced yield; however, raising it above this point resulted in a substantial decline in output.

**Fig. 1:**
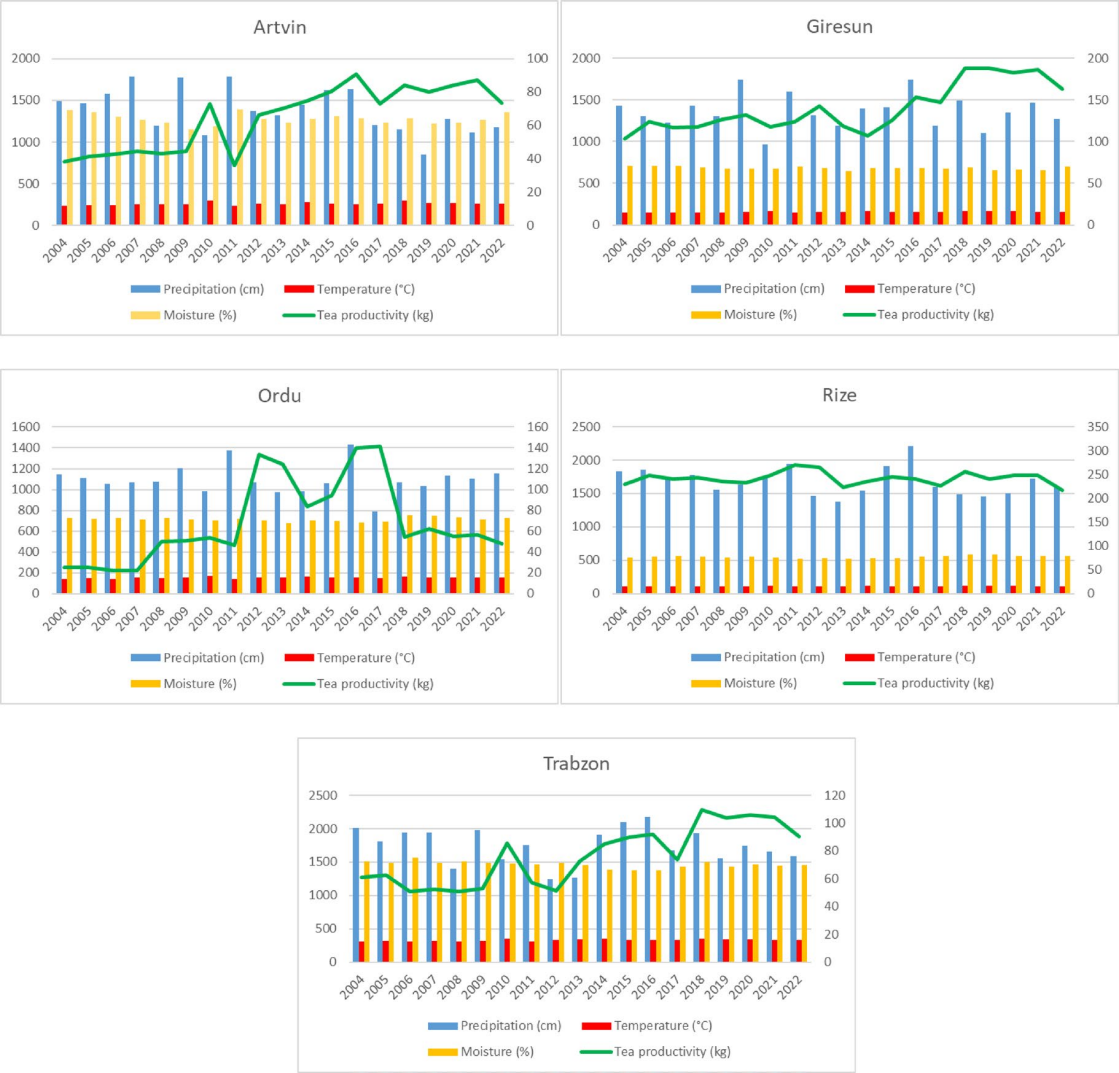
Climatic variables and tea productivity by provinces between 2004 and 2022 (left axis: tea productivity; right axis: climatic variables).

Despite these studies, significant gaps remain in understanding how climate change affects tea productivity, particularly for developing countries. In this regard, Bayraç and Doğan^56^ explored how climate change affected agricultural yield in Türkiye, but they did not provide empirical evidence that rising average temperatures have a detrimental impact on agriculture. Akcan et al.^57^ also used the autoregressive distributed lag (ARDL) limits test approach to examine how climate change affected the agriculture industry from 1985 to 2018 in Türkiye. The findings of the study demonstrate that rising temperature adversely affected the agricultural sector. Studies focusing on Türkiye, covering the period 1975–2019, have shown significant relationships between temperature and precipitation and tea yield; for example, İrdem^58^ analyzed tea yield in Türkiye between 1975 and 2019 and found a statistically significant positive correlation between annual tea yield and temperature variables such as maximum and minimum temperatures and annual precipitation level. Subsequently, the results of his study empirically demonstrate that while annual precipitation showed no significant relationship with tea yield, the effects of temperature were particularly pronounced during summer months. The findings also indicate that future temperature increases based on current climate change scenarios may further enhance tea productivity in the region. Similarly, Tutal^59^, using panel data analysis for the Eastern Black Sea region for the period 1970–2018, found that rising temperatures, particularly during the post-1993 “warming period,” have had a statistically significant positive effect on tea yield in Türkiye. In contrast, while precipitation showed a positive impact on yield prior to 1993, its influence diminished significantly in recent decades due to increased variability and extreme rainfall events, which may negatively affect soil structure and growing conditions. So, these results suggest to us that consistent warming has contributed to yield gains, whereas irregular precipitation patterns may pose emerging risks to tea cultivation in the region. In addition, Yazıcı^60^ highlights that the negative impacts of climate change on tea yield observed globally are also evident in Türkiye. Her study mainly emphasizes that these effects include irregular rainfall, elevated temperatures, shifts in harvest timing, increased incidence of pests and diseases, declining soil quality, and a notable rise in extreme weather events, all of which pose growing risks to sustainable tea cultivation. Beyond the empirical analysis, survey-based research conducted by Yıldız and Özcan^61^ also shows that shifting temperature averages, changing precipitation levels, and increasing rainfall irregularity have led to altitude-dependent impacts on tea production in this region. Their findings also demonstrate that while coastal lowland areas experience yield losses, particularly during the first flush harvest, higher-altitude regions tend to benefit from yield increases during the second and third harvests, which highlights a spatial differentiation in climate impacts across tea-growing zones. As shown in Fig. 1, tea productivity tends to increase in years with higher precipitation and temperature in the cities. However, relative humidity does not show a consistent link and decreases as yield increases. International systematic reviews and ecological suitability/modeling studies indicate that tea plants require high relative humidity, generally between 70% and 80%. However, excessive or prolonged humidity can reduce yield due to disease and physiological stress^62–64^.

Recent research across Asia and globally further underscores the diverse and region-specific impacts of climate change on tea production. For instance, Jayasinghe & Kumar^64^ documented that in Sri Lanka, moderate increases in temperature and rainfall can enhance yields, whereas prolonged droughts have adverse effects. Studies in China^54,65^ have linked drought frequency and heat waves to substantial interannual variability in tea output. Similarly, research from Kenya^55^ indicates that both short-term weather variability and long-term climate trends require adaptive cultivation strategies to maintain productivity. Comparative analyses, such as Ahmed et al.^19^ and Bania et al.^62^, suggest that while tea is sensitive to multiple climatic parameters, the magnitude, direction, and threshold levels of impacts vary substantially by region, which points to the importance of location-specific and data-driven adaptation policies.

Despite the increasing volume of empirical studies on the agricultural impacts of climate change, to the best of our knowledge, no prior research has systematically examined Türkiye’s tea sector through a dual empirical lens that combines the MMQR with advanced machine learning techniques. So, this study aims to fill this gap by combining MMQR and advanced machine learning techniques to offer novel insights into the climatic factors and their relation to tea productivity at the subnational level. This integrated approach allows for a more nuanced understanding of both distributional heterogeneity and non–linear interactions, offering innovative and policy-relevant insights into the climatic determinants of tea productivity at the subnational level.

## Model and variable specifications

The study explores the impact of climate change on tea productivity in provinces where tea has been cultivated in Türkiye between 2004 and 2022. We measured tea productivity (TP) in kilograms of tea per decare, and we quantified climate change with three key indicators: rainfall (PRE), mean temperature (TEM), and humidity (MOIS). Total tea area data is the tea production area (TPA). We sourced TP and TPA data from the Turkish Statistical Institute^66^, and we collected PRE, TEM, and MOIS data from the Turkish State Meteorological Service^67^. While these metrics capture general climate patterns, it is important to note that they represent mean climatic conditions rather than comprehensive climate change indicators. Climate change encompasses longer–term trends, increased variability, and the frequency of extreme events—dimensions not fully captured by annual averages alone. Nevertheless, it should be particularly emphasized that climate anomalies observed in parameters such as temperature, precipitation, and humidity are direct consequences of anthropogenic climate change, and variations in these parameters are regarded as manifestations of climate change itself.

The dataset consisted of annual observations for five provinces (Artvin, Giresun, Ordu, Rize, and Trabzon) over the period 2004–2022 (*N* = 5; T = 19). The primary reason for limiting the scope of the analysis to these five provinces was that commercial tea production in Türkiye was largely concentrated in them due to geographical and climatic conditions. In addition, the availability of long-term and consistent statistics provided by the Turkish Statistical Institute and the Turkish State Meteorological Service exclusively for the 2004–2022 period was a decisive factor in determining the duration of the analysis. Leng et al.^68^, Shan et al.^69^, Almulhim et al.^70^, and Chen et al.^71^, conducted with a similar panel data structure, employed the MMQR method in relatively small samples and obtained statistically significant and policy-relevant findings. This evidence demonstrated that the data structure and methodological choice of our study were consistent with the relevant literature. We applied a logarithmic transformation to minimize the effects of heteroskedasticity, balance the influence of outliers, and make the proportional relationships between variables more straightforward. Table 1 summarizes the description of the variables.

**Table 1:** Description of the variables.

Variable	Acronym	Definition (Unit)
Tea productivity	TP	It is calculated by dividing the total amount (kg) of tea by the total tea area (decare)
Precipitation	PRE	Total annual rainfall (Millimeter)
Temperature	TEM	Average annual temperature (Celsius)
Moisture	MOIS	Average annual relative moisture (%)
Tea production area	TPA	Total area of tea plantation (Decare)

Figure 1. visualizes the province–level temporal trends of climatic variables and tea productivity defined in Table 1 for the 2004–2022 period.

The estimating model of the study is constructed as in Eq. 1.

1 $$\:{{l}{n}{T}{P}}_{{i}{t}}={{{\beta}}_{0}+{{\beta}}_{1}{l}{n}{P}{R}{E}}_{{i}{t}}{+{{\beta}}_{2}{l}{n}{T}{E}{M}}_{{i}{t}}{+{{{\beta}}_{3}{l}{n}{M}{O}{I}{S}}_{{i}{t}}+{{{\beta}}_{4}{l}{n}{T}{P}{A}}_{{i}{t}}+{\varepsilon}}_{{i}{t}}$$

where *t* is the time dimension (*t* = 2004, …,2022), *i* is the cross-sectional dimension (*i* = 5), and $\:{\epsilon\:}$ is the error term. $\:{{\beta\:}}_{0}$ represents the constant term, while $\:{{\beta\:}}_{1}$, $\:{{\beta\:}}_{2}$, $\:{{\beta\:}}_{3}$, and $\:{{\beta\:}}_{4}$ are the explanatory variable parameters. $\:{{T}{P}}_{{i}{t}}$ represents the tea yield of province *i* in year *t*, while $\:{{P}{R}{E}}_{{i}{t}}$, $\:{{T}{E}{M}}_{{i}{t}}$, $\:{{M}{O}{I}{S}}_{{i}{t}}$, and $\:{{T}{P}{A}}_{{i}{t}}$ represent the total precipitation, average temperature, average relative humidity, and tea production area data for province *i* in year *t*, respectively.

## Methodology

### Panel data analysis

We perform some preliminary diagnostic tests before examining the basic estimation relationships among the variables in Eq. 1’s estimation model. Accordingly, the first stage is examining cross–section dependence (CSD), and the second stage is investigating the slope heterogeneity (SH) problems of the model. In panel data analysis, common shocks and similar external factors can lead to interdependence among cross-sectional units. In this context, controlling for CSD and selecting appropriate testing approaches that account for this phenomenon are of critical importance. The presence of CSD plays a decisive role in the choice of econometric methodology. Indeed, when CSD exists, more robust methods that explicitly account for such dependence should be employed instead of conventional testing approaches. Otherwise, neglecting CSD may result in biased and inconsistent estimates, misleading generalizations, and inappropriate policy recommendations. The relationships between variables may affect each unit—a country, region, or sector—to varying degrees. In this context, the assumption that slope coefficients are homogeneous across all cross units can lead to overlooking structural differences. Such an assumption may reduce the reliability of the estimated coefficients and weaken the validity of the model. The SH test is critically important for assessing whether the same policy instrument produces similar effects across different units. Based on the findings of the SH test, econometric methods that account for slope heterogeneity should be employed. Therefore, the control of CSD is conducted in detail using four different tests: CD of Pesaran^72^, CD_w_ of Juodis and Reese^73^, CD_w+_ of Fan et al.^74^, and CD* of Xie and Pesaran^75^. Then, we investigate the slope properties of the model using the CSD–sensitive Delta ($\:\widehat{\varDelta\:}$) and CSD–sensitive Adjusted Delta ($\:\widehat{\varDelta\:}$_adj_.) tests, newly proposed by Bersvendsen and Ditzen^76^.

In the third stage, we check the stationarity of the variables with the cross–sectionally IPS (CIPS) test developed by Pesaran and the multivariate augmented Dickey–Fuller (MADF) test introduced by Taylor and Sarno^77^, which considers both CSD and SH. In the fourth stage, we test for the presence of long–term relationships with cointegration tests based on Durbin Hausman (DH) of Westerlund^78^ and Lagrange Multiplier (LM) of Westerlund and Edgerton^79^. Both cointegration tests estimate reliable test statistics under SH and CSD conditions. The DH test adds flexibility to cointegration tests by allowing the explanatory variables to be I(0), provided that the dependent variable is I(1). In addition, this test offers two different test statistics: DH_panel_, which addresses slope homogeneity, and DH_group_, which pertains to slope heterogeneity. The LM test provides robust results even in small samples.

The fifth stage involves the estimation of long–run coefficients. The pool of quantile–based estimation techniques offers an array of options. Among these, we prefer the novel and robust MMQR approach developed by Machado and Silva^80^. Researchers looking into climate change, global warming, energy, and environmental issues have often used this method because (a) it gives more information than OLS–based methods that only look at average relationships; (b) it can handle outliers and heterogeneity; (c) it is based on covariance; (d) it gives accurate estimates even when there is multicollinearity and endogeneity; (e) it works well even with models that are not linear or normally distributed; f) While conventional econometric approaches may encounter efficiency and consistency issues in small sample sizes, the MMQR approach, with its moment-based structure, provides stronger and more reliable estimates; and g) Moreover, it enables researchers to analyse not only the mean or specific quantiles but also all quantiles within the Q_10_–Q_90_ range simultaneously. These features give the MMQR approach distinct advantages over traditional methods and other quantile–based estimation techniques, making it a suitable choice for this study. Equation 2 displays the basic form of the quantile regression Eq.

2 $$\:{{Q}}_{{{Y}}_{{i},{t}}}\left({\tau\:}/{{X}}_{{i},{t}\:}\right)={{\beta}}_{{\tau\:}}+{{X}}_{{i},{t}}^{{{\prime}}}{\beta}{{\alpha\:}}_{{\tau\:}}$$

In Eq. 2, $\:{{Q}}_{{{Y}}_{{i},{t}}}\left({\tau\:}/{{X}}_{{i},{t}\:}\right)$ represents the conditional quantile $\:{\tau\:}$^*th*^. In other words, it refers to the distribution of the dependent variable under a given quantile ($\:{\tau\:}$^*th*^). $\:{{\beta\:}}_{{\tau\:}}$ represents the effect of unobservable factors, whereas $\:{{\alpha\:}}_{{\tau\:}}$ acts as a predictor for the independent variable. *X*_*it*_ denotes the set of independent variables. *q(*$\:{\tau\:}$*)* denotes the sample quantile ($\:{\tau\:}$^*th*^) derived from the optimization problem. The definition of optimization is the same as in Eq. 3.

3 $$\:{{m}{i}{n}}_{{q}}\sum\nolimits_{{i}}\sum\nolimits_{{t}}{{p}}_{{\uptau}}({{R}}_{{i}{t}}-\left({{\delta}}_{{i}}+{{Z}}_{{i}{t}}^{{{\prime}}}{\gamma\:}\right){q})$$

Equation 4 illustrates the quantile regression model constructed within the framework of the Eq. 1 estimation model.

4 $$\:{{Q}}_{{T}{P}{i}{t}}\left({{\tau}}_{{k}\:}/{{{\beta}}_{{i}},{X}}_{{i}{t}\:}\right)={{\beta}}_{{i}}+{{\beta}}_{1{\tau}}{{P}{R}{E}}_{{i}{t}}+{{\beta}}_{2{\tau}}{{T}{E}{M}}_{{i}{t}}+{{\beta}}_{3{\tau}}{{M}{O}{I}{S}}_{{i}{t}}+{{\beta}}_{4{\tau}}{{T}{P}{A}}_{{i}{t}}$$

We employ in the sixth stage the panels corrected standard errors (PCSE) and feasible generalized least squares (FGLS) estimation tests to validate the MMQR model results as robustness tests. It is possible for these tests to take CSD and unobserved heterogeneity into account; they are also unaffected by possible autocorrelation and heteroscedasticity issues^81^.

### Ensemble learning

Ensemble learning algorithms combine multiple machine learning models to learn from the errors of weak learners and thereby generate stronger and more accurate predictions^82^. In this context, we employ the GB and XGBoost algorithms. GB uses gradient descent to minimize the loss function of the previous model, training new weak models by minimizing the gradients it computes at each stage. Each new model adds its predictions to the ensemble until it meets the stopping condition. XGBoost is an extended form of GB with superior speed, efficiency, and performance. Furthermore, XGBoost shows excellent performance and generalization ability in addressing nonlinear problems. Its most important feature is the use of L1 (lasso) and L2 (ridge) regularization to avoid overfitting. The steps of the XGBoost algorithm are (a) preprocessing the data; (b) making an initial tree model to find the starting point; (c) updating the model with GB; (d) finding the best values for the hyperparameters; (e) performing regularization to lower the risk of overfitting; and (f) building the final model and determining model performance values^83^.

For high prediction accuracy, it is important to determine the appropriate hyperparameter values for the methods. For this purpose, grid search or random search operations are useful. On the one hand, whereas a grid search systematically scans the hyperparameter space and tries all possible combinations, the computations are time-consuming for models with many hyperparameters. Random search, on the other hand, selects a random sample from the hyperparameter space, which may prevent finding the optimal combination and may not provide an exact solution. We estimate the hyperparameters of GB and develop a model for the hyperparameters of XGBoost with a grid search. In addition, we propose a new GA–XGBoost model by estimating it with a genetic algorithm based on heuristic algorithms.

**Fig. 2:**
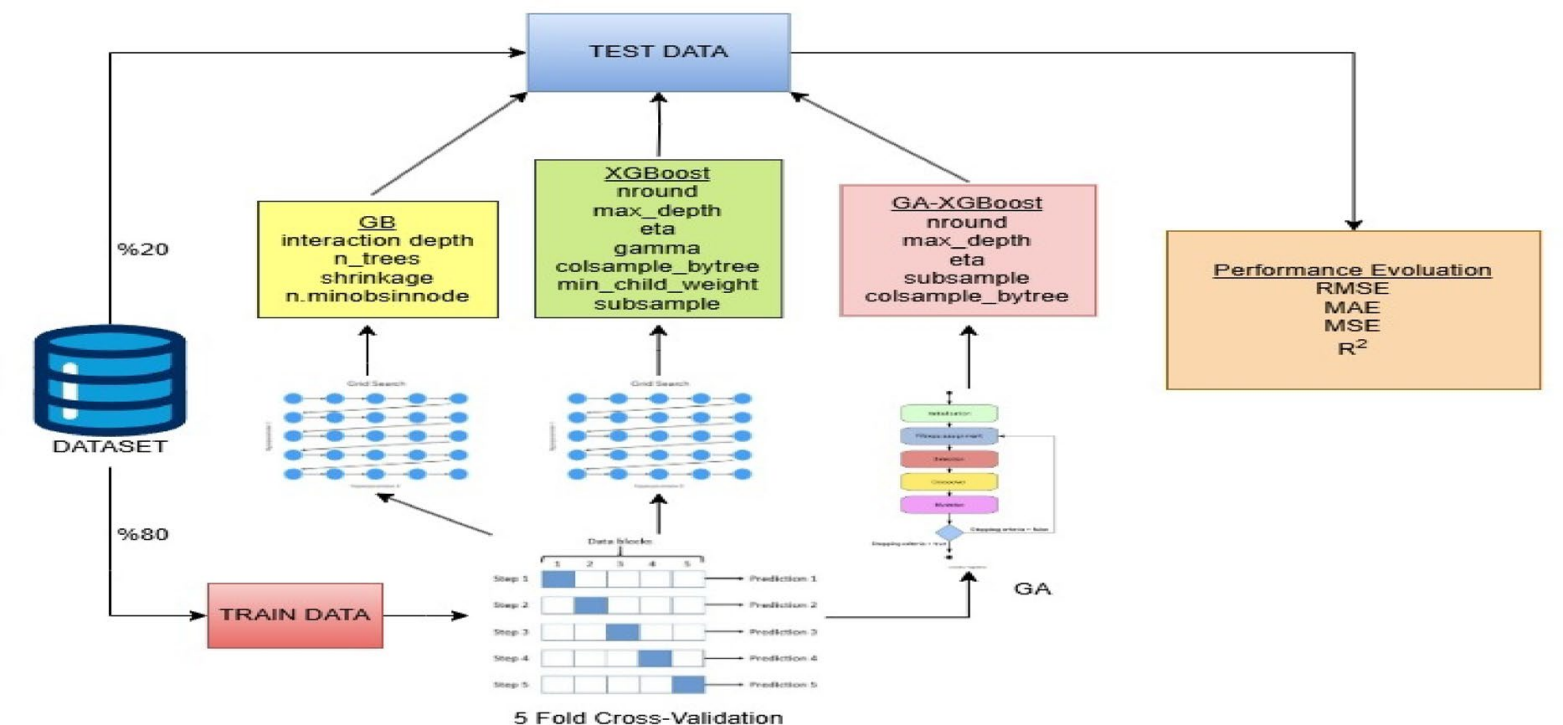
Proposed model.

We estimate the model using three different methods. First, we divide the dataset into two categories: training (80%) and testing (20%). We train the training data using the GB, XGBoost, and GA–XGBoost algorithms. We assess the test data by computing various performance criteria. We use a grid search to determine the hyperparameters of the GB and XGBoost algorithms. GA optimizes the hyperparameters of the GA–XGBoost algorithm. We apply a 5–fold validation for hyperparameter selection. We calculate root mean squared error (RMSE), mean absolute error (MAE), mean squared error (MSE), and coefficient of determination (R²) values to evaluate model performances. Figure 2 displays the roadmap for the machine learning algorithms used in this study.

**Fig. 3:**
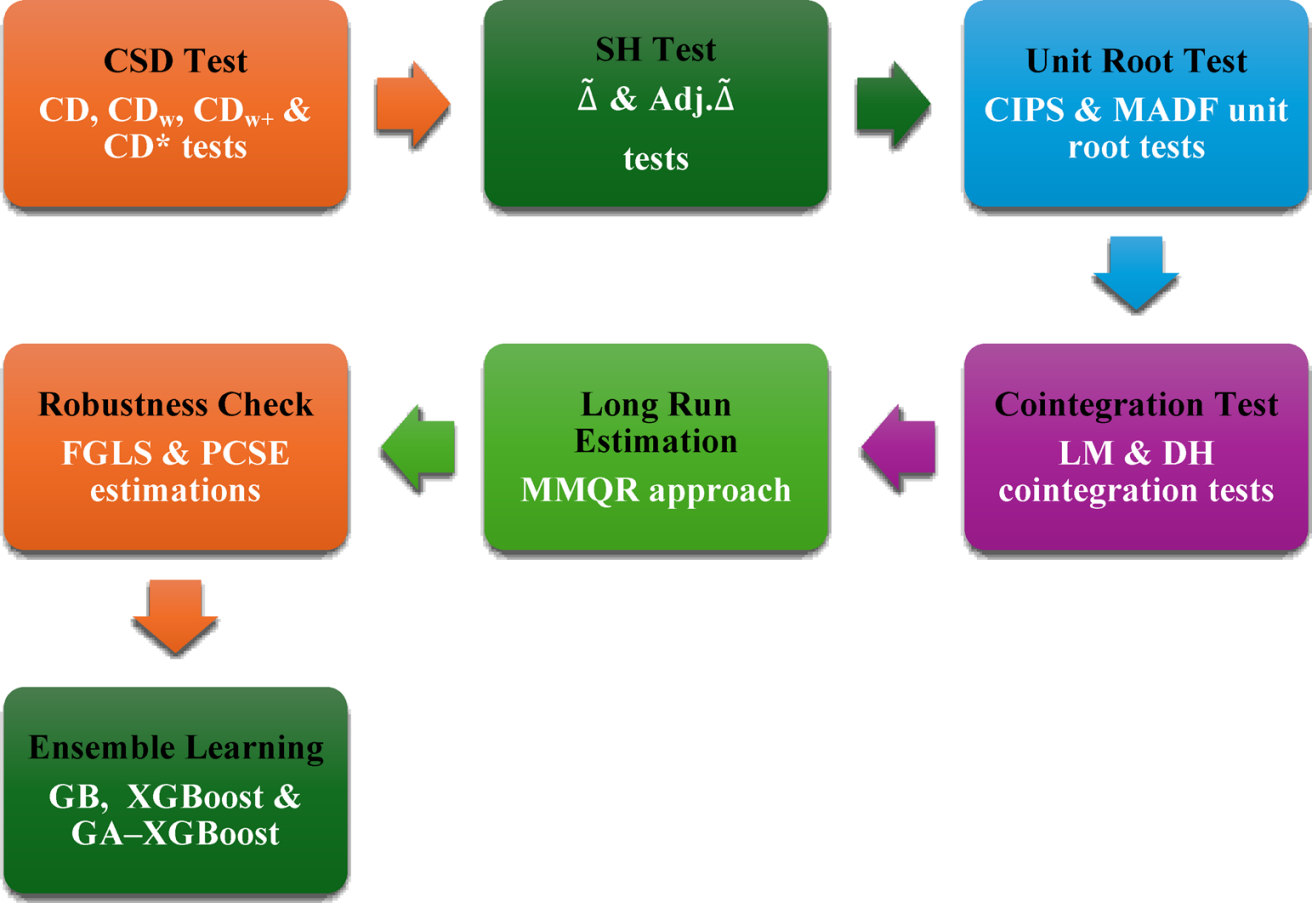
Flowchart of methodology.

Panel data analyses were performed using *Stata/MP 17.0* (StataCorp LLC, College Station, TX, USA), while all machine learning analyses were conducted using *R version 4.4.2* (R Core Team, 2024) in *RStudio version 2024.9.1.394* on a Windows 11 × 64 (build 26100) operating system. The primary R packages included gbm (2.2.2) for Gradient Boosting, xgboost (1.7.11.1) for Extreme Gradient Boosting, GA (3.2.4) for Genetic Algorithm optimization, and caret (7.0.1) for model training and cross-validation. All analyses are reproducible with the provided dataset and code specifications. Figure 3 summarizes the methodology of the study in a seven-stage flowchart.

## Results and discussion

As can be seen in Table 2’s descriptive statistics, the variables with the highest mean and median are TPA and TP, respectively. The variable with the highest maximum value is TPA, whereas the variable with the lowest minimum value is TEM. The variables with the highest and lowest standard deviation values are TPA and MOIS, respectively. In general, the distribution and variance characteristics of variables should be taken into account when choosing parametric or nonparametric tests. The Chen–Shapiro^84^ normal distribution test shows that all variables do not fit the normal distribution except the TEM variable, suggesting that quantile–based estimation methods that provide reliable and robust results should be preferred for data that do not meet the assumption of normal distribution.

**Table 2:** Descriptive statistics.

Variable	Type	Mean	Median	Std. Dev.	Min.	Max.	Chen-Shapiro
TP	Overall	7.097	7.264	0.515	5.412	7.733	0.922***
	Between			0.434	6.353	7.452	
	Within			0.336	6.155	7.995	
PRE	Overall	7.040	6.974	0.455	6.052	8.038	0.994**
	Between			0.478	6.522	7.756	
	Within			0.15	6.57	7.322	
TEM	Overall	2.706	2.727	0.087	2.442	2.833	0.960***
	Between			0.081	2.563	2.755	
	Within			0.048	2.586	2.825	
MOIS	Overall	4.248	4.252	0.071	4.055	4.405	1.008
	Between			0.069	4.155	4.345	
	Within			0.035	4.149	4.335	
TPA	Overall	10.178	11.360	3.034	3.807	13.231	0.899***
	Between			3.361	4.546	13.141	
	Within			0.274	9.439	11.193	

Note: ***p 0.01, **p 0.05.

In the first stage, we checked whether CSD exists. Table 3 displays the CSD results, which indicate that the null hypothesis, which assumes the absence of CSD, is largely rejected, and CSD is present in all variables. This suggests that taking CSD into account in the analysis is critical for the accuracy and reliability of the results.

**Table 3:** Cross-sectional dependence (CSD) test results.

Variables	CD	CDw	CDw+	CD
TP	4.68***	–1.97**	13.63***	–0.56
PRE	7.54***	1.01	24.84***	3.55***
TEM	12.85***	–2.65***	37.97***	0.73
MOIS	3.46***	0.68	11.88***	3.51***
TPA	–1.51	4.51***	32.45***	1.08

Note: ***p 0.01, **p 0.05.

In the second stage, we evaluate the slope homogeneity of the model parameters. For this purpose, Table 4 shows the SH test results. $\:\widehat{\varDelta\:}$ and $\:\widehat{\varDelta\:}$_adj_ test statistics reveal that the null hypothesis assuming slope homogeneity is rejected and the model coefficients exhibit heterogeneity by differing across units.

**Table 4:** Slope homogeneity (SH) test results.

	Blomquist and Westerlund (2013)		Bersvendsen and Ditzen (2020)	
Test	Test stat.	p–value	Test stat.	p–value
$\:\widehat{\varDelta\:}$	2.637***	0.008	–3.162***	0.002
$\:\widehat{\varDelta\:}$adj.	3.188***	0.001	–2.000**	0.046

Note: ***p 0.01, **p 0.05.

In the third stage, we analyze the stationarity properties of the variables. In this context, Table 5 shows the results of CIPS and MADF panel unit root tests. Both panel unit root test results indicate that all variables have a unit root at level, but all variables are stationary when first differences are taken.

**Table 5:** Unit root test.

Variables	CIPS test		MADF test	
	Level	1st difference	Level	1st difference
TP	–2.308	–3.308***	23.291	116.996***
PRE	–2.589	–4.561***	48.836	102.750***
TEM	–2.235	–3.207***	36.808	102.214***
MOIS	–2.382	–2.832***	27.382	79.896***
TPA	–1.484	–2.771***	13.506	102.213***

Note: ***p 0.01.

In the fourth stage, we investigate cointegration relationships. Table 6 presents the LM and DH cointegration test results. In the LM test, the bootstrap p-value is considered in the presence of CSD, whereas the asymptotic p–value is regarded in the absence of CSD. Therefore, we use the bootstrap p–value because CSD was found in the study. This result implies that we cannot reject the null hypothesis of cointegration’s nonexistence. In the DH test, the null hypothesis that there is no cointegration is rejected according to both test statistics, and cointegration exists.

**Table 6:** Cointegration test results.

LM test	Test statistic		Bootstrap *p*–value		Asymptotic *p*–value
	5.661		0.967		0.000

DH test	Test statistic		p–value		
DH_group_	2.493***		0.006		
DH_panel_	4.463***		0.000		

Note: ***p 0.01.

We estimate the long-term coefficients in the fifth stage. Table 7 reports the results of MMQR, which uses nine quantiles (Q = 0.10, 0.20, ., 0.90). The findings from the MMQR reveal heterogeneous effects of PRE, TEM, MOIS, and TPA on tea yield. In this context, the effect of PRE, TEM, MOIS, and TPA on tea yield varies significantly across cantiles. PRE exhibits a positive and significant effect on tea yield in all quantiles except Q_(0.80)_ and Q_(0.90)_. In contrast to higher quantiles, PRE coefficients show a significant relationship with tea yield at lower quantile levels. The PRE coefficient is relatively low at higher quantile levels (e.g., 0.184 at Q_(0.70)_) and comparatively high at lower quantile levels (e.g., 0.237 at Q_(0.10)_). These findings suggest that PRE plays a critical role in increasing tea yields in provinces with lower tea yields. These findings are consistent with the results of Ahmed et al.^19^ and Mallik and Ghosh^53^, who emphasized that PRE variability significantly affects tea production in India. On the other side, Wijeratne^51^ found that increases in temperature, soil moisture deficit, and saturation pressure deficit at low elevations in Sri Lanka can negatively impact tea productivity. This divergence may be due to differences in topography and soil structure between the different regions.

When we evaluate the situation in terms of PRE, the increase in the PRE observed in the Eastern Black Sea Region in recent years has a positive effect on tea yields. This effect reflects not only the increase in total precipitation but also the regional shifts in rainfall regimes and the restructuring of the hydrological cycle driven by climate change. In the Eastern Black Sea region, the rise in rainfall intensity and the sustained soil moisture over the past two decades have helped stabilize water availability for tea plants. Accordingly, the positive impact of precipitation on tea productivity can be interpreted as a beneficial outcome of climate change through its reinforcing effect on the regional water cycle.

The MMQR results of our study similarly show a positive and significant relationship between TEM and tea yield in all quantiles, emphasizing the important role of TEM in tea yields. In this context, TEM tends to have a greater impact on tea yield at lower quantiles (e.g., 2.672 at Q_(0.10)_) than at higher quantiles (e.g., 2.151 at Q_(0.90)_). This suggests that in provinces with lower tea yields, TEM is another critical factor in increasing those yields. We found that the average TEM changes observed at the regional level positively affected tea yield. On the axis of climate parameters, Cheserek et al.^55^, Yan et al.^65^, Lou et al.^54^, and Wu et al.^85^ suggested that TEM is one of the most important limiting parameters in tea cultivation. More importantly, this finding is broadly in line with the results of Lou et al.^54^, İrdem^58^, Tutal^59^, and Wu et al.^85^, who observed that moderate increases in average TEM can improve physiological functions in tea plants. However, our results differ from Biggs et al.^52^, who found that excessive heat in some Indian regions had a detrimental effect.

The MMQR results reveal that TEM exerts a strong and positive effect on tea yields across all quantiles. This outcome reflects the regional warming trend driven by anthropogenic climate change, which has enhanced photosynthetic efficiency and extended the growing season. The positive response indicates that current warming remains within the crop’s physiological tolerance, yielding short-term productivity gains. However, this effect is not purely climatic but a manifestation of ongoing climate change, as continued warming may push temperatures beyond optimal levels. Thus, temperature captures both the adaptive and risk dimensions of climate change in regional tea farming.

MMQR results also demonstrate a negative and significant link between MOIS and tea yield in all quantiles. These findings prove that MOIS has a negative and significant influence on tea yield. In this context, the effect of MOIS on tea yield is stronger and more negative in higher quantiles (e.g., − 3.189 at Q_(0.90)_), whereas the effect is lower in lower quantiles (e.g., − 2.451 at Q_(0.10)_). The findings of this study, which reveal the negative effect of MOIS on tea yield, are consistent with the results obtained by Bania et al.^62^, Shrestha and Miles^63^, and Jayasinghe and Kumar^64^. The MMQR results indicate that MOIS has a negative and statistically significant effect on tea yields across all quantiles. This finding reflects the adverse impacts of anthropogenic climate change, which has intensified regional humidity levels and increased their variability. Global warming enhances the atmosphere’s water-holding capacity and, combined with altered rainfall and temperature patterns, causes persistently high relative humidity in the Eastern Black Sea region. Such conditions exceed the tea plant’s optimal humidity range (≈ 70–80%), leading to respiratory stress, greater fungal disease incidence, and yield loss. Overall, the stronger negative effect in higher quantiles indicates that climate change–induced humidity pressure poses a growing challenge for sustainable tea farming in high-yield areas.

Finally, the results show that the effect of the control variable TPA on tea yield is positive and significant across all cantiles. These findings indicate that the effect of TPA on tea yield decreases as we move towards higher quantiles, but this effect increases significantly towards lower quantiles. It reveals that the expansion of tea farming areas, especially in low-yielding provinces, has a stronger and more significant effect on tea yields than in high-yielding provinces. This suggests that soil diversity in large areas in the region may increase tea yields. The findings of this study, which indicate that TPA has a positive and significant effect on TP, are consistent with the results of studies by Bania et al.^62^ and Shrestha & Miles^63^.

**Table 7:** Method of moments quantile regression (MMQR).

Variables	Quantiles								
	Q_(0.10)_	Q_(0.20)_	Q_(0.30)_	Q_(0.40)_	Q_(0.50)_	Q_(0.60)_	Q_(0.70)_	Q_(0.80)_	Q_(0.90)_
PRE	0.237* (0.138)	0.214** (0.098)	0.204** (0.088)	0.196** (0.084)	0.191** (0.084)	0.184** (0.087)	0.180** (0.091)	0.163 (0.114)	0.156 (0.126)
TEM	2.672*** (0.627)	2.521*** (0.448)	2.458*** (0.401)	2.406*** (0.383)	2.377*** (0.383)	2.332*** (0.397)	2.304*** (0.413)	2.194*** (0.517)	2.151*** (0.570)
MOIS	–2.451** (1.061)	–2.665*** (0.755)	–2.754*** (0.677)	–2.828*** (0.648)	–2.869*** (0.648)	–2.933*** (0.670)	–2.972*** (0.697)	–3.128*** (0.875)	–3.189*** (0.968)
TPA	0.203*** (0.027)	0.162*** (0.022)	0.144*** (0.019)	0.130*** (0.017)	0.122*** (0.016)	0.110*** (0.018)	0.102*** (0.019)	0.072*** (0.023)	0.060*** (0.022)
Constant	6.107* (3.477)	8.243*** (2.501)	9.135*** (2.240)	9.877*** (2.130)	10.285*** (2.119)	10.922*** (2.208)	11.317*** (2.292)	12.871*** (2.866)	13.485*** (3.151)

Notes: ***p 0.01, **p 0.05, *p 0.1. Standard errors (S.E) are in parentheses.

**Fig. 4:**
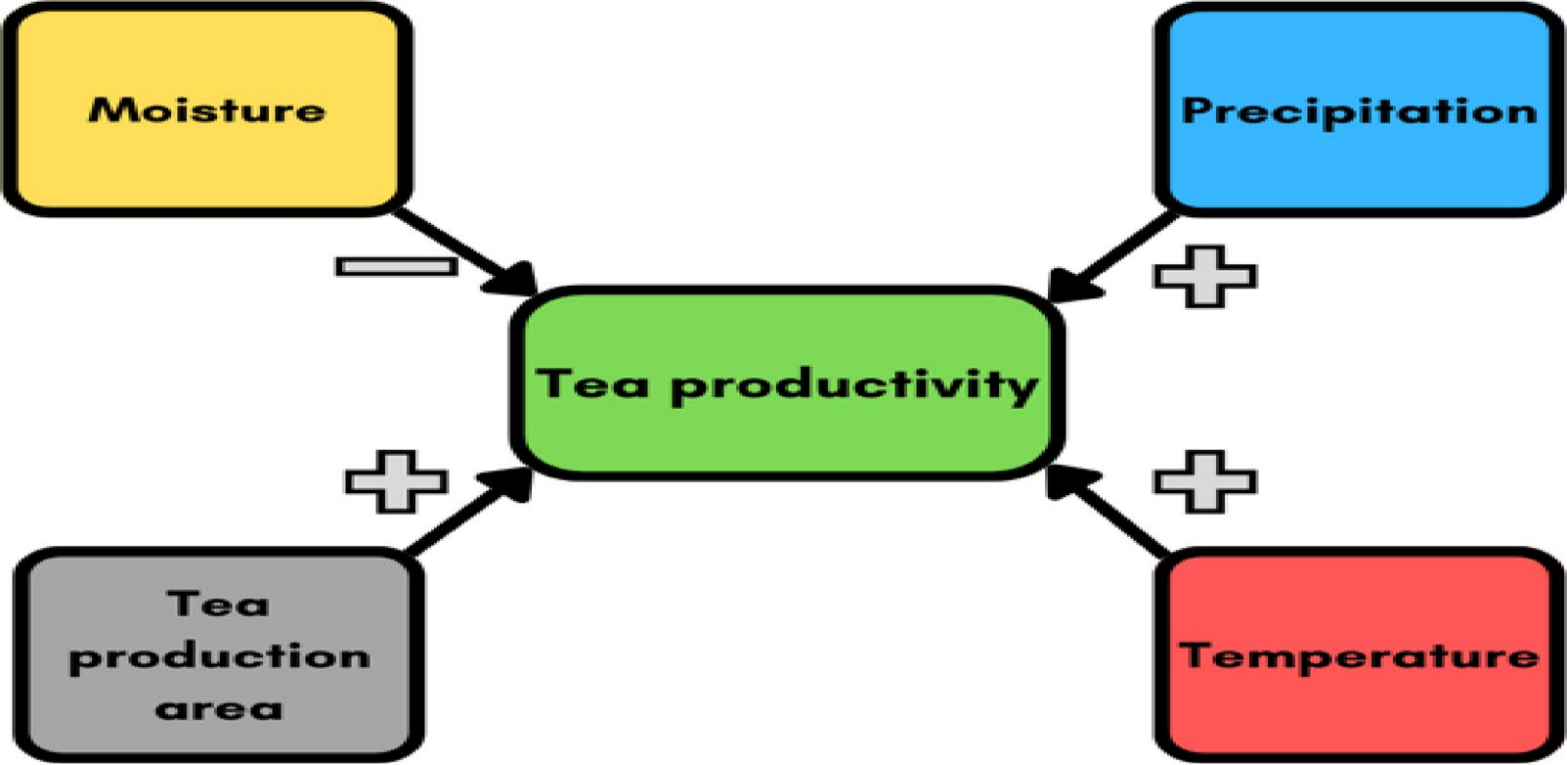
Graphical summary of MMQR, FGLS, and PCSE estimation findings.

Moreover, in the sixth stage of the analysis, we check the robustness of the MMQR results using PCSE and FGLS regression estimations. Table 8 presents the FGLS and PCSE estimation results. PRE, one of the variables affecting tea yield, shows a positive and significant effect, supporting the claim that an increase in rainfall can increase tea yields. Similarly, the TEM variable also has a positive and significant relationship. Thus, we find that temperature is a positive and significant variable for tea yield. In contrast, the MOIS variable has a negative and significant effect on tea yield. This indicates that humidity may be a risk factor, especially for sustainable tea farming. In addition, the TPA variable also exhibits a positive and significant relationship. In this context, expanding agricultural areas increases tea yield. These results are consistent with the findings from the MMQR analysis and support the strong link between tea yield and climate change indicators. Figure 4 provides a graphical summary of the empirical findings.

**Table 8:** FGLS and PCSE Estimation results.

Variable	FGLS	PCSE
PRE	0.177*** (0.060)	0.194** (0.078)
TEM	1.300*** (0.226)	2.395*** (0.389)
MOIS	–1.642*** (0.322)	–2.843*** (0.556)
TPA	0.107*** (0.016)	0.127*** (0.016)
Constant	8.234*** (1.227)	10.025*** (1.611)
Wald χ2	81.91*** (0.000)	69.52*** (0.000)

Notes: ***p 0.01, **p 0.05, *p 0.1. Standard errors (S.E) are in parentheses.

In addition to panel data analysis, in the seventh stage, we use GB, XGBoost, and GA–XGBoost from ensemble learning algorithms in a different methodological approach. For GB hyperparameters, we use “interaction depth” for maximum depth, “n.trees” for number of trees, “shrinkage” for learning rate, and “n.minobsinnode” for minimum number of leaf nodes. For the hyperparameters of XGBoost, “nround” indicates how many trees the model will be updated with, “max_depth” specifies the maximum depth of the tree, “eta” represents the learning rate, “gamma” is the minimum error required for a split to occur, “colsample_bytree” refers to the number of feature subsets used, “min_child_weight” denotes the minimum number of samples needed to create a leaf node, and “subsample” indicates the sampling rate. Table 9 shows the tested and assigned values for the hyperparameters.

**Table 9:** Tested and selected hyperparameter values for the methods.

Method	Hyperparameters	Tested Values	Assigned Value
GB	interaction depth	1,3,5	5
	n_trees	50,100, 150	100
	shrinkage	0.01, 0.1, 0.3	0.3
	n.minobsinnode	5, 10, 20	5
XGBoost	nround	50, 100, 150	150
	max_depth	3, 5, 7	3
	eta	0.01, 0.1, 0.3	0.1
	gamma	0, 1, 5	0
	colsample_bytree	0.6, 0.8	0.6
	min_child_weight	1, 3, 5	3
	subsample	0.6, 0.8	0.8

The GA–XGBoost method determined the hyperparameter values for both GA and XGBoost. For XGBoost, we optimized values in the range of nround 50–500, max_depth 3–10, eta 0.01–0.3, subsample, and colsample_bytree 0.5–1.0 with GA. The maximum number of iterations for GA is set to 50, with a crossover rate of 0.8 and a mutation rate of 0.1. Table 10 presents the model performance values for various population sizes.

**Table 10:** Model performance values.

Population Size	RMSE	MAE	MSE	R2
20	0.0939	0.0773	0.0088	0.8938
30	0.1064	0.0766	0.0113	0.8635
40	0.1012	0.0808	0.0102	0.8767
50	0.1027	0.0798	0.0105	0.8730
60	0.0970	0.0777	0.0094	0.8867

According to the RMSE values, the population size for the best performance value is 20. Therefore, we determined the optimal parameter values for the GA–XGBoost algorithm to be nround 323, max_depth 6, eta 0.1, subsample 0.8, and colsample_bytree 0.8. In this study, we looked at the GB, XGBoost, and GA–XGBoost algorithms. Table 11 shows the efficacy of the models and the importance of the variables.

**Table 11:** Variable importance levels according to GB, XGBoost and GA–XGBoost methods.

Variables	GB	XGBoost			GA-XGBoost		
	Importance Score	Gain	Cover	Frequency	Gain	Cover	Frequency
TPA	39.816 (1)	0.512 (1)	0.281	0.279	0.386 (1)	0.338	0.249
PRE	22.972 (2)	0.175 (3)	0.193	0.196	0.249 (2)	0.229	0.309
TEM	17.550 (4)	0.132 (4)	0.234	0.218	0.227 (3)	0.221	0.240
MOIS	19.66 (3)	0.179 (2)	0.290	0.305	0.136 (4)	0.210	0.200
RMSE	0.123	0.112			0.093		
MAE	0.094	0.088			0.077		
MSE	0.015	0.012			0.008		
R2	0.816	0.846			0.893		

According to the model performance criteria (min RMSE, MAE, MSE, and max R²), the GA–XGBoost method performed best. TPA stood out as the variable with the highest significance, whereas the MOIS was the least significant variable. Although empirical analyses revealed that the MOIS had a negative and significant effect on tea yield, machine learning models identified this variable as the least important factor. The result indicates that the effect of humidity on tea yield may be complex. Moreover, this indicates that humidity is less determinative than other climatic and environmental factors, emphasizing that strategies to increase tea production and yields in the region should focus on changes in more influential parameters such as temperature, precipitation, and agricultural areas.

## Conclusion and policy implications

Climate change has had significant negative impacts on agricultural productivity, reducing capacity to meet the food needs of a growing population. In this context, increasing tea productivity, one of the most consumed beverages and strategic agricultural products on a global scale, is critical for agricultural sustainability and reducing economic inequalities. Therefore, conducting an in–depth analysis of the abiotic (soil quality and climate variables) and biotic factors (pests, diseases, weeds, and plant competition) affecting tea cultivation and determining the necessary climatic parameters for sustainable tea farming is of great importance. Given this critical need, this study empirically examined the impact of climate change on tea yields in Türkiye’s tea–growing regions between 2004 and 2022 by using both MMQR and machine learning methods. So, this dual methodological approach enhances the robustness of our findings and brings new perspectives on climate adaptation in tea agriculture.

Empirical results revealed that precipitation and average temperature had a positive effect on tea productivity. At the same time, whereas the results also indicated that increased humidity had a negative impact on tea productivity, machine learning models showed that humidity was a lower determinant than other climatic and environmental factors. This suggested that although monitoring humidity was necessary for sustainable tea production, it would be more strategic to prioritize more influential parameters such as temperature, precipitation, and agricultural area. In addition, the expansion of the tea cultivation area had a positive impact on tea productivity. These results prove that policies aiming to increase productivity in tea production should be reevaluated within the framework of climate change, land use, and sustainable agricultural practices. Based on these results, we provide several key policy recommendations to ensure sustainable tea production in Türkiye, with a particular focus on adapting to climate change and improving agricultural practices.

- Because Artvin, Giresun, Ordu, Rize, and Trabzon are the provinces where tea is intensively cultivated in Türkiye, policymakers should promote climate–adapted agricultural techniques such as drip irrigation and soil moisture conservation methods in this region. As a policy justification for this point, we consider that these techniques may directly address the identified need for efficient water management and protection against extreme weather conditions, which are projected to become more frequent due to climate change.
- Although expanding the area under tea cultivation increases yields, increasing the productivity of existing agricultural land offers a more sustainable solution. Problems such as excessive moisture and erosion negatively affect soil fertility, especially in Rize and Trabzon. Therefore, policymakers should focus on promoting soil conservation measures, fertilization based on soil analysis, and the use of erosion control techniques, such as further green cover through afforestation. Subsequently, we believe that these kinds of actions directly address agricultural production and soil degradation, which is a key concern highlighted by the study, and align with global calls for more sustainable agricultural practices under changing climate conditions.
- Early warning systems that are region–specific and sensitive to climatic change would ensure that farmers are prepared for sudden weather events (floods, hail, excessive rainfall) or long–term expected weather conditions. Accessible channels such as mobile applications, local radio and television broadcasts, and social media platforms could deliver the needed information to farmers.
- Because traditional agricultural methods have a negative impact on tea yields, farmers should use innovative agricultural products such as sensors, drones, and IoT–based irrigation systems in the region. In this context, grants and low-interest loans can help farmers access innovative agricultural technologies, and training programs should be organized for their use. So, we believe that these technologies can increase efficiency and sustainability in tea farming by addressing the identified challenges of soil and water management under changing climate conditions.
- Policymakers should prioritize reducing farmers’ input costs and enabling them to better market their products, as well as promoting and strengthening tea producer cooperatives. In addition, joint strategies to combat climate change can arise from regional cooperation.

This study examines the relationship between tea yield and climate change in Türkiye’s Eastern Black Sea Region using an integrated methodological framework combining MMQR and machine learning models (GB, XGBoost, GA–XGBoost). The proposed approach is readily transferable to other countries, regions, and agricultural contexts where comparable climate and production data are available. This framework enables researchers to analyze heterogeneous effects across yield distributions while identifying critical determinants of agricultural productivity. However, given substantial regional variations in climate conditions, soil characteristics, and farming practices, country-specific calibration and validation are essential for ensuring result reliability. Beyond its academic contribution, this transferable framework provides policymakers with a replicable analytical tool for developing evidence-based, location-specific agricultural and climate adaptation strategies.

This study has some limitations. First, care should be taken when generalizing the findings to other countries or regions with different climatic or geographical environments, as this study only covers the provinces of Artvin, Giresun, Ordu, Rize, and Trabzon in Türkiye. While the scope of this study offers valuable regional insights, it may not capture the broader variability in tea production practices worldwide. Moreover, the dataset used in this study covers the period from 2004 to 2022, which may not fully account for long-term climate-induced impacts or cyclical climatic patterns. Notably, the reliance on annual aggregated data limits our ability to capture intra-annual variability and extreme weather events that may critically affect tea yield. Furthermore, the study primarily examines climate variables such as precipitation, temperature, and humidity, while explicitly excluding other crucial factors such as soil quality (e.g., pH, organic matter, nutrient availability, soil structure, soil moisture), agricultural technologies, and land management practices, etc., which may also significantly influence tea productivity. The absence of soil quality data is particularly significant, as soil fertility, structure, and erosion patterns are known to directly affect tea plant growth and yield sustainability in the Eastern Black Sea Region. Additionally, topographical variables such as altitude, slope, and aspect, as well as biotic factors including pest pressure and disease incidence, were not incorporated due to data unavailability, limiting a more holistic understanding of tea productivity determinants. In this context, future research could address these limitations by expanding the dataset to include longer time periods, higher temporal resolution (e.g., monthly or seasonal data), more diverse geographical locations, soil quality parameters, topographical variables and biotic factors. Future studies can conduct more comprehensive analyses by incorporating environmental, economic, and socioeconomic variables such as soil, land, production techniques, biotic and abiotic factors, input costs, farmer income, and agricultural subsidies that affect tea yield, using high-frequency data, spatial models, and machine learning techniques.

## Supplementary Information

Below is the link to the electronic supplementary material.

Supplementary Material 1

## Data Availability

This study used publicly available data and needed no informed consent. Tea production and agricultural area data were obtained from the Turkish Statistical Institute (https://biruni.tuik.gov.tr/medas), and climate change data (precipitation, temperature, and humidity) were retrieved from the Turkish State Meteorological Service (https://www.mgm.gov.tr/). All datasets used in the analysis are cited and described in detail within the manuscript.

## Code availability

All custom R scripts used for the machine learning analyses, as well as all Stata scripts used for the coefficient estimation procedures, are available at: 10.5281/zenodo.17610824.
