# Supplementary material for: Assessing climate change effects on Turkish tea farming through a dual approach using MMQR and machine learning
Source: Sci Rep. 2025 Dec 16;16:122. doi: 10.1038/s41598-025-29358-8 (PMC12764820; doi:10.1038/s41598-025-29358-8)
Supplement: Supplementary file 1 — Supplementary Material 1 [file 41598_2025_29358_MOESM1_ESM.docx]

Appendix

Table A1. Diagnostic test results

| Diagnostic test | Test statistic | p-value | Result |
| --- | --- | --- | --- |
| Variance inflation factor (VIF) | Mean VIF= 1.67 |  | There is no multicollinearity |
| VIF > 10 or 1/VIF < 0.1 | 1/VIF ranges between 0.457 and 0.918 |  |  |
| Breusch–Pagan/Cook–Weisberg test | chi^2^(1)=52.01 | 0.000 | Presence of heteroscedasticity |
| Wooldridge test for autocorrelation  in panel data | F(1, 4)=109.151 | 0.000 | There is first-order autocorrelation |
| Durbin–Wu–Hausman  test | chi^2^(1)=0.011 | 0.918 | There is no endogeneity |
